# Real-Time Two-Dimensional Magnetic Particle Imaging for Electromagnetic Navigation in Targeted Drug Delivery

**DOI:** 10.3390/s17092050

**Published:** 2017-09-07

**Authors:** Tuan-Anh Le, Xingming Zhang, Ali Kafash Hoshiar, Jungwon Yoon

**Affiliations:** 1 School of Mechanical and Aerospace Engineering & ReCAPT, Gyeongsang National University, Jinju 660-701, Korea; tuananhle@gnu.ac.kr; 2 School of Naval Architecture and Ocean Engineering, Harbin Institute of Technology at Weihai, Weihai 264209, China; zhxm@hit.edu.cn; 3 Faculty of Industrial and Mechanical Engineering, Islamic Azad University, Qazvin Branch, Qazvin 34199-15195, Iran; hoshiar@GNU.ac.kr; 4 School of Integrated Technology, Gwangju Institute of Science and Technology, 123 Cheomdan-gwagiro, Buk-gu, Gwangju 61005, Korea

**Keywords:** magnetic particle imaging, navigation resolution, magnetic nanoparticles

## Abstract

Magnetic nanoparticles (MNPs) are effective drug carriers. By using electromagnetic actuated systems, MNPs can be controlled noninvasively in a vascular network for targeted drug delivery (TDD). Although drugs can reach their target location through capturing schemes of MNPs by permanent magnets, drugs delivered to non-target regions can affect healthy tissues and cause undesirable side effects. Real-time monitoring of MNPs can improve the targeting efficiency of TDD systems. In this paper, a two-dimensional (2D) real-time monitoring scheme has been developed for an MNP guidance system. Resovist particles 45 to 65 nm in diameter (5 nm core) can be monitored in real-time (update rate = 2 Hz) in 2D. The proposed 2D monitoring system allows dynamic tracking of MNPs during TDD and renders magnetic particle imaging-based navigation more feasible.

## 1. Introduction

Magnetic nanoparticles (MNPs) are effective drug carriers that can deliver the optimal dose of a drug to a targeted region deep in the body, and show promise for treating severe illnesses. Despite the significant improvements in modern drug design [1,2], the required targeting precision is lacking, which gives rise to undesirable side effects. Thus, magnetic drug delivery (MDD) systems have been developed to add drugs to MNPs and then apply magnetic fields for concentrating them at disease locations, such as solid tumors, regions of infection, or blood clots [3,4]. Precise steering of nanocarriers in a vascular system, however, remains a major challenge.

To achieve high-performance microparticle steering, multicoil-magnetic systems were developed [5,6,7]. The idea of using a field function (FF) to steer nanoparticles and solve particle-vessel sticking issue was introduced in [8]. Moreover, the FF concept was improved to include the aggregated particle phenomenon [9]. Although these results allow MNPs to target the brain [10], the suggested open-loop approach does not guarantee a high targeting efficiency of MDD systems due to its deficiency for the monitoring of MNPs.

Several feedback methods have been proposed for targeted drug delivery (TDD), such as using ultrasound to locate micro-size particles [11] or a microscope for visual tracking [12]. The groundbreaking concept of magnetic resonance navigation (MRN), which includes a feedback control scheme, was proposed by Martel et al. [13]. MRN gains benefits from using a Magnetic Resonance Imaging (MRI) system, which is well developed and highly stable for human scale applications and successfully has been applied for actuation and tracking of microparticles [14,15,16,17]. However, the magnetic field gradient for MRI systems is limited to a maximum of 400 mT/m/μ_0_, which is not sufficient for remote control of an untethered device in human fluids [18] and the MNPs [19]. 

Magnetic particle imaging (MPI) is a fast and sensitive imaging modality that enables measurement of the spatial distribution of MNPs [20]. MPI systems have millimeter-scale spatial resolutions and high temporal resolutions [20]. Tracer MNPs in MPI systems can provide spatial information and may be used as drug carrier particles. The high temporal and spatial resolution of MPI can fulfill the requirements of cardiovascular, neurologic, and peripheral vascular applications [21]. Moreover, the high gradient field over 1 T/m/μ_0_ enables the nanoparticle-based drug delivery to a mouse brain [10]. In MPI, the detection threshold of magnetic tracers is less limited by background signals from the host tissue compared to MRI [22]. Thus, an image reconstruction method of MPI can be simpler than MRI. However, since MPI compared to MRI is a more recent monitoring system, it needs further studies for human scalability. 

Therefore, a real-time navigation system for MNPs based on MPI is capable of performing actuation and monitoring tasks simultaneously. This allows precise steering and tracking of MNPs with a high efficiency for TDD. A novel MPI-based navigation system that enables steering of MNPs by combining the electromagnetic actuator (EMA) mode and the MPI mode was introduced in [23]. A one-dimensional (1D) MPI-based navigation system was developed for simultaneous monitoring and actuation of MNPs [24,25]. Despite the improvements in real-time monitoring systems for steering MNPs, however, such systems are limited to 1D monitoring and steering. Even though it is difficult to apply the MPI mode for a feedback-based steering control of MNPs in a vascular network, it can allow a user to monitor MNPs reaching to a desired region inside the body. A monitoring function for the positions of MNPs can positively affect the success rate in drug delivery. In addition, it can help optimizing an feed-forward control scheme [8,10], which is being applied to current drug delivery systems due to its simplicity of realization.

In this paper, we introduce a real-time two-dimensional (2D) MPI monitoring scheme for 2D navigation of MNPs. This paper is organized as follows: in Section 2, the 2D MNP navigation system is introduced. In Section 3, the 2D signal model and image reconstruction of amplitude-modulation MPI (AM MPI) is developed. The 2D AM MPI experimental results are presented in Section 4. Section 5 provides a conclusion and discussion of future works.

## 2. Two-Dimensional MNP Navigation System

**Figure 1:**
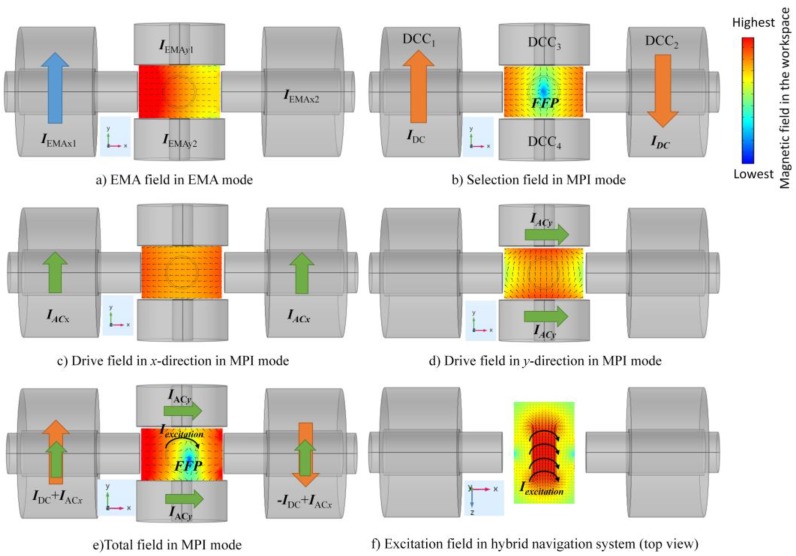
The functional magnetic fields generated by different currents in the workspace. The color and length of the cones represent the magnitude field strength, while the direction of the cones denotes the field direction. (**a**) During the electromagnetic actuator (EMA) period, the EMA field, which is a gradient field used to steer the magnetic nanoparticles (MNPs) to the left-side of workspace, is induced by current *I*_EMAx1_ in differential-current coil 1 (DCC_1_). (**b**) A field-free point (FFP) is located at the center of the selection field, which is generated by Maxwell coil pair (Both coils in Maxwell coil pair are applied with the same *I*_DC_ current but different coil direction. (**c**,**d**) The drive fields in magnetic particle imaging (MPI) mode are generated by time-varying drive fields with *I*_ACx_ and *I*_ACy_ currents and *f_x_* and *f_y_* frequencies in the *x*- and *y*-axes coils, respectively. (**e**) Total field results in MPI mode. Movement of the FFP depends mainly on the selection field and the drive fields (*I*_DC_ and *I*_AC_ are applied simultaneously), and the FFP moves in the *x*-*y* plane. To oscillate the magnetization of the particles, an excitation field is generated by the excitation coil with a high-frequency and low-amplitude *I_excitation_* current. The amplitude of the excitation field is small enough to ignore FFP movement in the *z*-axis. The excitation field is homogeneous, as shown in (**f**).

In previous works [24,25], we introduced a hybrid navigation system consisting of an electromagnetic actuation (EMA) mode and an MPI mode. The coil setup for the 2D navigation system is shown in Figure 1. 

In EMA mode, the differential-current coils (DCC) [19] are used to load the *I*_EMAx1_, *I*_EMAx2_ and *I*_EMAy1_, *I*_EMAy2_ currents for generation of the magnetic field gradient in the workspace, which steers the MNPs towards the current-carrying coil. By controlling the amplitude of the current within the four coils, we can control the direction of movement of MNPs. For example, to move MNPs to the left side of an *x*-axis, the current *I*_EMAx1_ is applied to the DCC_1_ coil of the *x*-axis, as shown in Figure 1a.

In MPI mode, two DCCs (DCC_1_ and DCC_2_) in the *x*-axis are loaded with currents, *I_DC_*, which have equal amplitude but opposite directions to generate the selection field and a field-free point (FFP) (Figure 1b). Movement of the FFP in 2D is achieved by superposition of the static selection field and two time-varying drive fields; the drive fields are induced by the *I*_ACx_ and *I*_ACy_ currents and the *f_x_* and *f_y_* frequencies in the *x*- and *y*- axes coils, respectively. The two drive fields are spatially homogeneous (Figure 1c,d). By combining the low-frequency drive fields and the selection field through one set of coils (Figure 1e), the FFP is moved in the field-of-view (FOV) plane. In this paper, the Cartesian trajectory is used for the FFP trajectory. The size of the Cartesian trajectory depends on the amplitude of *I*_ACx_ and *I*_ACy_. The frequencies of drive fields will decide the density of the Cartesian trajectory [26]. An excitation field of frequency *f_E_* is employed to oscillate the magnetization of the particles and generate the signal, as shown in Figure 1f.

Real-time MPI and EMA in the proposed navigation system can be achieved using a time-division multiplexing scheme to the coil topology [24,25], which combines the EMA and MPI modes via time sequencing. A navigation period includes at least one MPI scanning period, to detect the position of MNPs, and one EMA period to control their positions. In EMA mode, the magnetic force is sufficiently high to steer MNPs; however, it also saturates them. Therefore, to start MPI mode, the MNPs must be demagnetized. Moreover, the currents in coils cannot be changed immediately between EMA and MPI modes due to coil impedance. For these reasons, a relaxation period is necessary to demagnetize the MNPs and prevent a voltage surge from the coils, the relaxation time prolongs the navigation time slightly (0.05 s) [25]. In MPI mode, the alternating magnetic field may affect the position of the MNPs; however, our previous work confirmed that the magnetic force during the MPI cycle has little effect on the position of MNPs compared to that in EMA mode [23], because the drive fields create only an average force that has a value of zero [27]. Therefore, MPI mode operates during off-cycle periods in the actuation duty cycle for navigation control.

**Figure 2:**
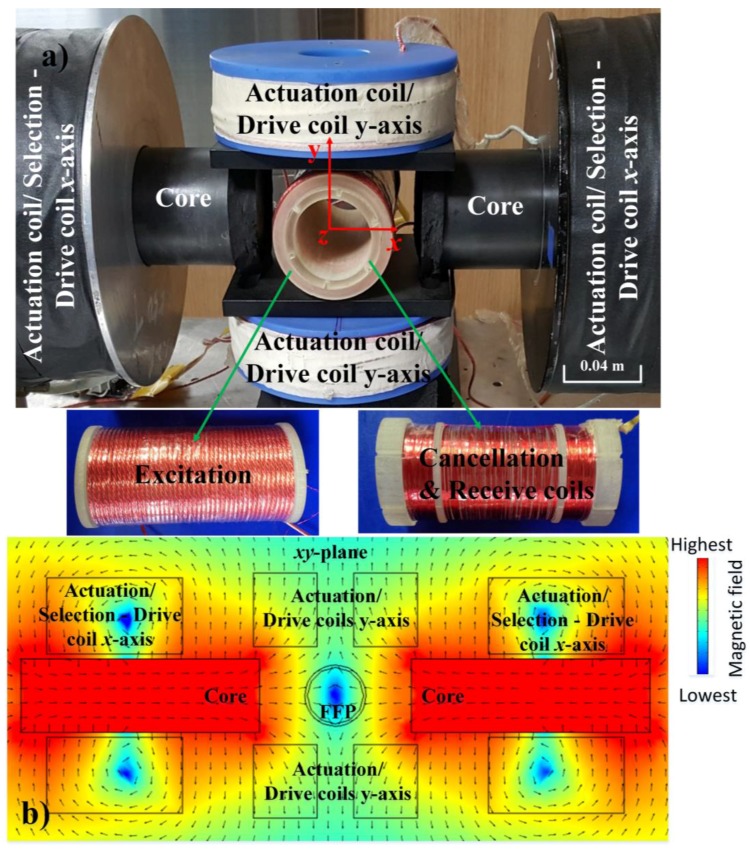
Experimental setup for two-dimensional (2D) navigation of MNPs. (**a**) Coil configuration setup in the 2D navigation system experiment. (**b**) Animation of FFP used for 2D magnetic particle imaging (MPI) signal generation is illustrated in the *x*-*y* plane. The color and length of the cones represent the magnitude of the field, while the direction of the cones indicates the field direction.

To increase the resolution of the proposed 2D AM MPI system, which will be explained in the next section, iron cores are added to the *x*-axis coil to increase the magnetic force in the *x*-axis. The center of the signal-receive coil is defined as the coordinate origin, the axes of the DCCs are the *x*- and *y*-axes, and the axis of the receive coil is the *z*-axis. The coil configuration setup in the navigation system experiment is shown in Figure 2.

## 3. Two-Dimensional AM MPI Signal and Image Reconstruction

In previous works [24,25], we theorized a 1D amplitude modulation (AM) MPI signal imaging process. The particle signal can be described as a sinusoidal voltage under an excitation frequency that has amplitude as a convolution. In this section, we can extend the 1D AM MPI theory into two dimensions (2D), provided that the particle signal is similar to the 1D method. The AM MPI method can overcome difficulties for 3D extension of existing MPI schemes [26,28] since the proposed scheme requires only one cancellation coil and one receive coil for 3D. Moreover, the AM MPI requires only a narrow-band receive coil for easy impedance matching and elimination of noises from other frequencies, and use of a low-amplitude excitation field can reduce the likelihood of unpleasant peripheral nerve stimulation (PNS) [29]. For image reconstruction, a simple interpolation method can be used for real time applications. Besides, the utilizing cores in the system enables higher magnetic gradient, which is essential for nanoparticle-based delivery [19]. 

### 3.1. Generation of an FFP and Its Movement in the FOV

A time-independent magnetic field, known as a selection field and generated by *I_DC_*, is applied to DCC_1_ and DCC_2_ to obtain an FFP for locating the scanning points. The selection field relies on a 3D linear gradient in the form:

(1) $$H_{s}\left(r\right)=Gr=G_{x}\left(\begin{array}{l} 1\;0\;0\\ 0\;-\frac{1}{2}\;0\\ 0\;0\;-\frac{1}{2} \end{array}\right)\left(\begin{array}{l} x\\ y\\ z \end{array}\right)=\left[\begin{array}{l} G_{x}x\\ G_{y}y\\ G_{z}z \end{array}\right],$$

where the vector $r={\left[x\text{​ }y\;z\right]}^{T}$ denotes position in real space and *G_x_* = −2*G_y_* = −2*G_z_* are magnetic field gradient in *x*, *y* and *z* directions, respectively. The MPI spatial resolution is anisotropic and twofold greater in the *x*- versus the *y*- and *z*-directions, due to the fundamentally anisotropic magnetic field gradient.

In addition to the selection field, we can add an orthogonal set of homogeneous magnets that produce static and time-varying fields to shift the FFP by applying *I_ACx_* to DCCs in the *x*-axis and *I_ACy_* in the *y*-axis. Then, the total drive fields are:

(2) $$H^{D}\left(t\right)=H_{x}^{D}\left(t\right)+H_{y}^{D}\left(t\right)=\left[\begin{array}{l} -A_{x}^{D}\cos\left(2\pi f_{x}t\right)\\ -A_{y}^{D}\cos\left(2\pi f_{y}t\right)\\ 0 \end{array}\right]=\left[\begin{array}{l} H_{Dx}\left(t\right)\\ H_{Dy}\left(t\right)\\ 0 \end{array}\right],$$

where: $A_{x}^{D},A_{y}^{D}$ is the amplitude of the drive field in the *x*- and *y*-directions, respectively. *f_x_* and *f_y_* denote frequencies in *x*, *y*-axes coils, respectively, and satisfy a constraint *f_y_* >> *f_x_*.

The drive field in the *x*-axis and *y*-axis are:

(3) $$H_{x}^{D}\left(t\right)=H_{Dx}\left(t\right)e_{x}=-A_{x}^{D}\cos\left(2\pi f_{x}t\right)e_{x},\;H_{y}^{D}\left(t\right)=H_{Dy}\left(t\right)e_{y}=-A_{y}^{D}\cos\left(2\pi f_{y}t\right)e_{y},$$

where ***e****_x_*, ***e****_y_* are unit vector in the *x*- and the *y*-axes, respectively.

### 3.2. Low-Amplitude, High-Frequency Excitation Field

To oscillate the magnetization of the particles, a high-frequency excitation field *f_E_* >> *f_x_*, *f_y_* with a low-amplitude $A_{z}^{E}$ is created by applied *I*_excitation_ to the excitation coil. The excitation field is:

(4) $$H^{E}\left(t\right)=H_{Ez}\left(t\right)e_{z}=-A_{z}^{E}\cos\left(2\pi f_{E}t\right)e_{z},$$

where ***e****_z_* is a unit vector in the *z*-axis. 

The amplitude $A_{z}^{E}$ of excitation field must be small enough to ignore the moving FFP in the *z*-axis ($A_{z}^{E}>>A_{x}^{D},A_{y}^{D}$).

### 3.3. Total Magnetic Field

From Equations (1), (2) and (5), the total magnetic field can be described as:

(5) $$H\left(r,t\right)=H^{D}\left(t\right)+H^{E}\left(t\right)+H_{s}=H^{DE}\left(t\right)+H_{s}=\left[\begin{array}{l} H_{x}\left(x,t\right)\\ H_{y}\left(y,t\right)\\ H_{z}\left(z,t\right) \end{array}\right],$$

where: the total field of the drive fields and excitation fields is:

(6) $$H^{DE}\left(t\right)=H^{D}\left(t\right)+H^{E}\left(t\right),$$

the total magnetic field in the *x*-, *y*- and *z*-axes are:

(7) $$H_{x}\left(x,t\right)=H_{Dx}\left(t\right)+G_{x}x,\;H_{y}\left(y,t\right)=H_{Dy}\left(t\right)+G_{y}y,\;H_{z}\left(z,t\right)=H_{Ez}\left(t\right)+G_{z}z,$$

and the current utilized in the coils are:

(8) $$I_{1x}=I_{DC}+I_{ACx}\cos(2\pi \;f_{x}t),\;I_{2x}=-I_{DC}+I_{ACx}\cos(2\pi \;f_{x}t),\;I_{1y}=I_{ACy}\cos(2\pi \;f_{y}t),\;I_{2y}=I_{ACy}\cos(2\pi \;f_{y}t),$$

From Equation (5), we can solve the instantaneous location of the FFP, ***r****^FFP^*(*t*) such that ***H***(***r***, *t*) = 0:

(9) $$r^{FFP}\left(t\right)=-G^{-1}H^{DE}\left(t\right),$$

Then, the magnetic field at an arbitrary point ***r*** is related to the instantaneous position of the FFP:

(10) $$H\left(r,t\right)=G(r^{FFP}\left(t\right)-r),$$

### 3.4. Magnetization of Particles

The MPI signal relies on the nonlinearity of the MNP magnetization curves, with the MNP magnetization saturated with the saturation field strength. The relationship between the magnetization of the particles and the external magnetic field can be described using the Langevin theory of paramagnetism [20]:

(11) $$M\left(H\right)=cmL\left(\beta \left\|H\right\|\right)\hat{H},$$

where *L* is the Langevin function, in particular *L*(0) = 0, *m* is the magnetic moment of a single magnetic nanoparticle, *c* is the concentration of particles, ***H*** is the vector of the applied magnetics field, $\hat{H}$ is the direction vector of ***H***, and $\beta =\frac{\mu_{0}m}{k_{B}T_{P}}$ is a property of the magnetic particle; *k_B_* is the Boltzmann constant, and *T_p_* is the particle temperature. For a spherical particle, the magnetic moment can be computed as *m* = *M*_sat_(π/6)*d*^3^, where *M*_sat_ = 0.6 T/μ_0_ is the saturation magnetization for magnetite and *d*[m] is the particle diameter.

### 3.5. Signal Produced by 2D AM MPI

Consider a continuous distribution of magnetic nanoparticles with density *c*(***r***). From Equations (10) and (11), we can rewrite the magnetization of nanoparticles located at ***r****^FFP^*(*t*) position as follows:

(12) $$M\left(r,t\right)=c\left(r\right)mL\left[\beta \left\|G(r^{FFP}\left(t\right)-r)\right\|\right]\frac{G(r^{FFP}\left(t\right)-r)}{\left\|G(r^{FFP}\left(t\right)-r)\right\|},$$

Assuming a set sensitivity homogeneous receive coils, which has a constant sensitivity ***p***(***r***) = [*p_x_ p_y_ p_z_*]*^T^*, the induced voltage in three axes can be written in the form:

(13) $$u^{P}\left(t\right)=-\mu_{0}\frac{d}{dt}∭p\left(r\right)\partial M\left(r,t\right)d^{3}r,$$

To calculate the Equation (13), we define [30]:

(14) $$\left\{ \begin{array}{l} H\left(r,t\right)=\left[\begin{array}{l} H_{x}\left(x,t\right)\\ H_{y}\left(y,t\right)\\ H_{z}\left(z,t\right) \end{array}\right]\\ \hat{H}=\frac{H}{\left\|H\right\|} \end{array}\right.\Rightarrow \left\{ \begin{array}{l} \dot{H}={\dot{H}}^{DE}\left(t\right)\\ \dot{\hat{H}}=\frac{\dot{H}}{\left\|H\right\|}-\frac{{\dot{H}}^{T}H}{{\left\|H\right\|}^{3}}H \end{array}\right.,$$

The derivative of the quasi-static Langevin function with vector-valued, time varying operand ***H*** is given by:

(15) $$\frac{d}{dt}L\left(\left\|\beta H\right\|\right)\hat{H}=\dot{L}\left(\left\|\beta H\right\|\right)\beta \left(\frac{\dot{H}\cdot H}{{\left\|H\right\|}^{2}}H\right)+\frac{L\left(\left\|\beta H\right\|\right)}{\left\|H\right\|}\left(\dot{H}-\frac{\dot{H}\cdot H}{{\left\|H\right\|}^{2}}H\right),$$

By developing the Equation (15), we have:

(16) $$\frac{d}{dt}L\left(\left\|\beta H\right\|\right)\hat{H}=\left[\begin{array}{l} \frac{L\left(\beta \left\|H\right\|\right)}{\left\|H\right\|}\left({\dot{H}}_{Dx}-\frac{H_{xyz}H_{x}}{{\left\|H\right\|}^{2}}\right)+\frac{\dot{L}\left(\beta \left\|H\right\|\right)\beta }{{\left\|H\right\|}^{2}}H_{xyz}H_{x}\\ \frac{L\left(\beta \left\|H\right\|\right)}{\left\|H\right\|}\left({\dot{H}}_{Dy}-\frac{H_{xyz}H_{y}}{{\left\|H\right\|}^{2}}\right)+\frac{\dot{L}\left(\beta \left\|H\right\|\right)\beta }{{\left\|H\right\|}^{2}}H_{xyz}H_{y}\\ \frac{L\left(\beta \left\|H\right\|\right)}{\left\|H\right\|}\left({\dot{H}}_{Ez}-\frac{H_{xyz}H_{z}}{{\left\|H\right\|}^{2}}\right)+\frac{\dot{L}\left(\beta \left\|H\right\|\right)\beta }{{\left\|H\right\|}^{2}}H_{xyz}H_{z} \end{array}\right]=\left[\begin{array}{l} L_{x}\\ L_{y}\\ L_{z} \end{array}\right],$$

where: $H_{xyz}=\left(H_{x}{\dot{H}}_{Dx}+H_{y}{\dot{H}}_{Dy}+H_{z}{\dot{H}}_{Ez}\right)$

From Equation (16), we can rewrite the MPI signal in Equation (13) as:

(17) $$\begin{array}{l} u^{P}\left(t\right)=-\mu_{0}\frac{d}{dt}∭p\left(r\right)mc\left(r\right)L\left(\left\|\beta H\right\|\right)\hat{H}d^{3}r=-\mu_{0}∭p\left(r\right)mc\left(r\right)\left[\begin{array}{l} L_{x}\\ L_{y}\\ L_{z} \end{array}\right]d^{3}r\\ =\left[\begin{array}{l} -\mu_{0}∭p^{x}mc\left(r\right)L_{x}d^{3}r\\ -\mu_{0}∭p^{y}mc\left(r\right)L_{y}d^{3}r\\ -\mu_{0}∭p^{z}mc\left(r\right)L_{z}d^{3}r \end{array}\right]=\left[\begin{array}{l} u^{Px}(t)\\ u^{Py}(t)\\ u^{Pz}(t) \end{array}\right] \end{array},$$

where *u^Px^*(*t*), *u^Py^*(*t*), *u^Pz^*(*t*) are the particle signals measured in *x*, *y* and *z* directions, respectively. 

In our method, only the *z*-axis has a receive coil, so the particle signal measured by AM MPI is:

(18) $$\begin{array}{l} u^{PR}(t)=u^{Pz}(t)=-\mu_{0}∭p^{z}mc\left(r\right)L_{z}d^{3}r\\ =-\mu_{0}p^{z}∭mc\left(r\right)\frac{L\left(\beta \left\|H\right\|\right)}{\left\|H\right\|}{\dot{H}}_{Ez}d^{3}r-\mu_{0}∭p^{z}mc\left(r\right)\left(\frac{\dot{L}\left(\beta \left\|H\right\|\right)\beta }{{\left\|H\right\|}^{2}}-\frac{L\left(\beta \left\|H\right\|\right)}{{\left\|H\right\|}^{3}}\right)H_{xyz}H_{z}d^{3}r\\ =u^{P1}\left(t\right)+u^{P2}\left(t\right) \end{array},$$

where the *u^P^*^1^(*t*) is a major-component of the particle signal *u^PR^*(*t*):

(19) $$u^{P1}\left(t\right)=-\mu_{0}p^{z}∭mc\left(r\right)\frac{L\left(\beta \left\|H\right\|\right)}{\left\|H\right\|}{\dot{H}}_{Ez}d^{3}r,$$

and the *u^P^*^2^(t) is a minor-component of the particle signal *u^PR^*(*t*):

(20) $$u^{P2}\left(t\right)=-\mu_{0}∭p^{z}mc\left(r\right)\left(\frac{\dot{L}\left(\beta \left\|H\right\|\right)\beta }{{\left\|H\right\|}^{2}}-\frac{L\left(\beta \left\|H\right\|\right)}{{\left\|H\right\|}^{3}}\right)H_{xyz}H_{z}d^{3}r,$$

Because $A_{z}^{E}>>A_{x}^{D},A_{y}^{D}$ and *f_E_* >> *f_x_*, *f_y_* and $A_{z}^{E}f_{E}>>A_{x}^{D}f_{x},A_{y}^{D}f_{y}$. So $u^{P1}\left(t\right)>>u^{P2}\left(t\right)$.

Thus, Equation (18) can be rewritten as:

(21) $$u^{PR}(t)\approx u^{P1}()t=-\mu_{0}p^{z}∭mc\left(r\right)\frac{L\left(\beta \left\|H\right\|\right)}{\left\|H\right\|}{\dot{H}}_{Ex}d^{3}r,$$

This can be represented as a spatial convolution interrogated at the instantaneous FFP location:

(22) $$u^{PR}(t)\approx -\mu_{0}p^{z}mc\left(r^{FFP}(t)\right)***\frac{L\left(\beta \left\|Gr^{FFP}(t)\right\|\right)}{\left\|Gr^{FFP}(t)\right\|}A_{z}^{E}2\pi f_{E}\sin\left(2\pi f_{E}t\right),$$

### 3.6. Signal Processing

The voltage measured in the receive coil is a superposition of the particle signal *u^PR^*(*t*) induced by the time-varying magnetization and the excitation signal *u^E^*(*t*) induced by the time-varying magnetic field; i.e.,:

(23) $$u\left(t\right)=u^{PR}(t)+u^{E}\left(t\right),$$

The particle signal *u^PR^*(*t*) is considerably smaller than the induced excitation signal *u^E^*(*t*). The analog-to-digital converter (ADC) is not capable of resolving the combined signals, so the excitation signal needs to be removed from the received signal before using ADC [31]. To remove the excitation signal, we use a cancellation coil [28]. The cancellation coil and the receive coil have the same geometric parameters but different coil polarities. They are connected in series. The voltage measured in the cancellation coil *u^C^*(*t*) and the excitation signal *u^E^*(*t*) have the same amplitudes but opposite directions (or *u^E^*(*t*) ≈ −*u^C^*(*t*)). Then, the particle signal *u^PR^*(*t*) can be measured by combining the signals of the receive coil and the cancellation coil, as follows:

(24) $$u(t)=u^{PR}(t)+u^{E}(t)+u^{C}(t)\approx u^{PR}(t),$$

**Figure 3:**
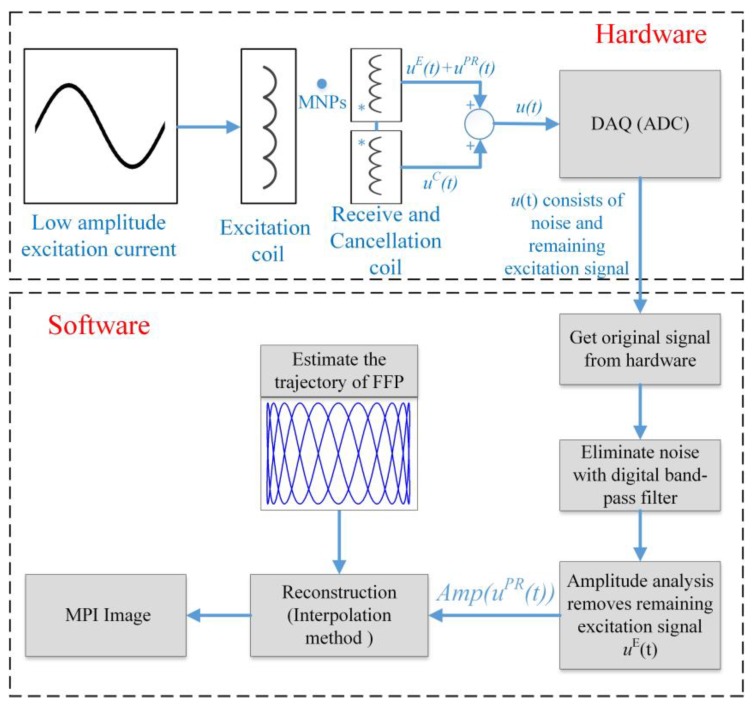
Signal flow diagram of the realized set-up.

The combined setup of the receive coil and cancellation coil can be seen in Figure 2. Using this combination, the ADC is capable of resolving the particle signal. Although the excitation signal *u^E^*(*t*) had been removed by the cancellation coil, in practice, the receiver signal after Equation (24) still contains noise because the excitation signal *u^E^*(*t*) cannot be deleted entirely. To receive only the particle signal *u^PR^*(*t*) after ADC, noise must be filtered-out using a digital band-pass filter with a higher order. Then, we continue to remove the remaining part of the excitation signal *u^E^*(*t*) by comparing the maximum and minimum magnitude of the signal through the received signal amplitude of the particle signal $Amp\left[u^{PR}(r^{FFP}\left(t\right))\right]$ of Equation (26), which will be explained in the next sub-section. This step is termed amplitude analysis, and is shown in Figure 3. 

### 3.7. Reconstruction of 2D AM MPI

Equation (22) shows that the received particle signal is a sinusoidal voltage with a frequency *f_E_*, and an amplitude proportional to the particle concentration at the FFP and the amplitude and frequency of the excitation field. Equation (22) also shows that the suggested AM MPI requires the amplitude of the received signal to reconstruct the MPI images. This signal will be used for image reconstruction.

According to Equation (22), the amplitude of the signal contains information on the particle concentration. The amplitude of the signal is given by:

(25) $$Amp\left[u^{PR}(r^{FFP}\left(t\right))\right]=-\mu_{0}p^{z}mc\left(r^{FFP(t)}\right)***\frac{L\left(\beta \left\|Gr^{FFP}(t)\right\|\right)}{\left\|Gr^{FFP}(t)\right\|}A_{z}^{E}2\pi f_{E},$$

This signal combined with the trajectory of the FFP can be used to reconstruct an image of the MNPs by an interpolation method [32]. The resulting image is given by:

(26) $$IMG(r^{FFP}\left(t\right))=Amp\left[u^{PR}(r^{FFP}\left(t\right))\right],$$

The image is updated every 0.5 s. The image reconstruction loop (hardware and software) lasts 0.5 s, and a dynamic 2D image can be generated. This 2D image could facilitate real-time monitoring of MNP steering in a feedback loop. A signal processing and reconstruction procedure for determining the particle concentration is shown in Figure 3.

## 4. Results and Discussion

Nanorobotics systems for TDD have been introduced as open-loop systems with a feedforward design for steering MNPs in Y-shaped vessels. The design has been examined by means of in vivo and in vitro studies [8,9]. Hybrid 1D monitoring and steering was introduced in [24,25]. For a feasible hybrid steering approach, a 2D real-time monitoring scheme for feedback is essential. The AM MPI concept has been utilized to design a nanorobotics system for real-time monitoring.

### 4.1. Experimental Setup

**Figure 4:**
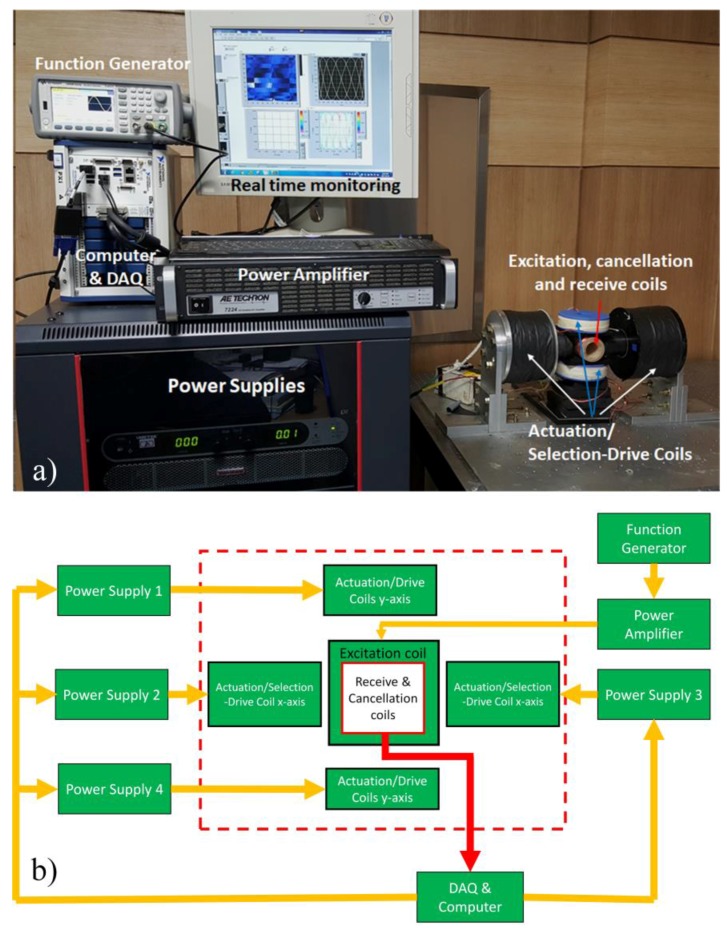
Two-dimensional real-time MNP imaging system. (**a**) System setup for 2D real-time MPI-based monitoring, (**b**) wiring scheme for the 2D MPI system.

The experimental setup of the 2D MNP monitoring system for the MPI-based nanorobotics platform is shown in Figure 4a. The FFP is generated using two selection-drive coils in the *x*-axis. Two cores to increase the magnetic field density (Vacoflux 50 [cobalt-iron alloy]; Vacuumschmelze, Hanau, Germany) are used on the *x*-axis; each is 0.195 m in length and 0.06 m in diameter. The specifications of the coils are shown in Table 1.

To change the position of the FFP, sinusoidal currents with different frequencies are used. The frequency in the *x*-direction (*f_x_*) is 4 Hz, and that in the *y*-direction (*f_y_*) is 26 Hz. Four DC power supplies (SGA 600/17; 10 kW, Ametek, Berwyn, PA, USA) are utilized to generate the sinusoidal currents. A sinusoidal signal with a high frequency (40 kHz) and low amplitude (200 μT/μ_0_), which is generated by a function generator (33500B Series, Keysight, Santa Rosa, CA, USA), is amplified by a power amplifier (7224, AE Techron, Elkhart, IN, USA), and the current is fed to the excitation coil. The hardware connections are shown in Figure 4b.

**Figure 5:**
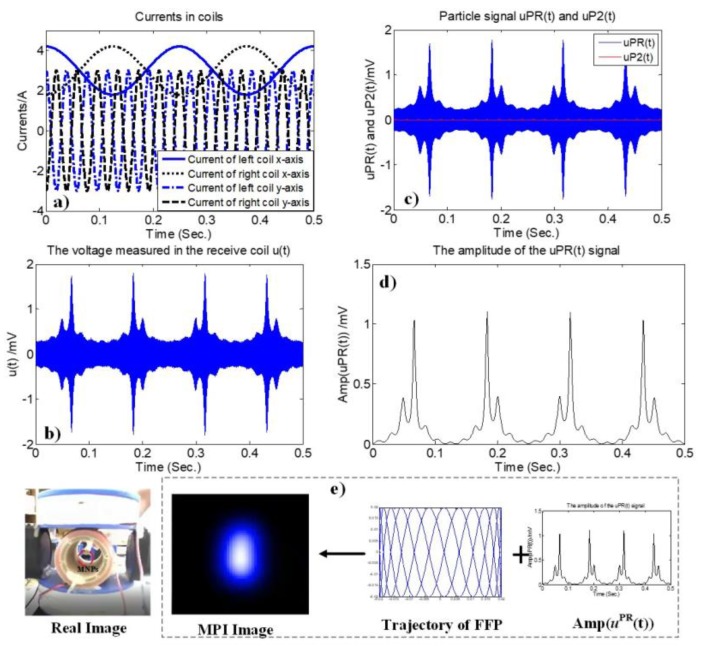
Two-dimensional amplitude-modulation (AM) MPI signal results for MNPs at the center of workspace with the following parameters: *t*_MPI_ = 0. 5 s, *I*_DC_ = 3 A, *I*_ACx_ = 1.2 A, *f_x_* = 4 Hz, *I*_ACy_ = 3 A, *f_y_* = 26 Hz; (**a**) waveforms of the currents in the DCCs in Equation (8). (**b**) Voltage measured in the receive coil *u*(*t*), (**c**) *u^PR^*(*t*) and minor-component *u^P^*^2^(*t*) in Equation (20) after noise removal by a band-pass filter, (**d**) amplitude of *u^PR^*(*t*) after the analysis phase, and (**e**) real and MPI images of MNPs at the center of workspace.

To assess 2D real-time AM MPI monitoring, the MNPs used were 45 to 65 nm in diameter with a 5 to 6 nm core size (Resovist; Meito Sangyo Co., Ltd., Tokyo, Japan). The 25 μL Resovist particles were poured into two tubes (Disposable Culture Tubes, Borosilicate Glass, MagQu, Taiwan of 6 mm × 50 mm size). These tubes were put together to make a sample for the experiment in Figure 5. The Fe content and magnetic susceptibility of the undiluted sample were ~58.6 mg/mL and 0.035 erg∙Gauss∙g^−1^, respectively, and the saturation magnetization value was 340 ± 10 kA/m [33]. The navigation system can monitor the positions of MNPs in real-time MPI images. To verify the real-time monitoring results, the positions of the MNPs were also determined using an optical camera.

### 4.2. Two-Dimensional Real-Time AM MPI Monitoring

The MNPs were placed along the FFP scanning trajectory in the *x*-*y* plane so that their concentration distribution could be monitored using 2D AM MPI. The MNP concentration was estimated from the amplitude of the received signal. The MNP sample was initially placed at the bottom right-side of the receive coil; the MNPs then move clockwise, and vice versa. The color spectrum (from white to black) is utilized to reconstruct the MPI images. Pure black indicates the absence of MNPs and pure white indicates the highest concentration of MNPs. The maximum AM MPI signals are thought to indicate the positions of the MNPs. The 2D AM MPI signal results for MNPs at the center of workspace are shown in Figure 5. 

**Figure 6:**
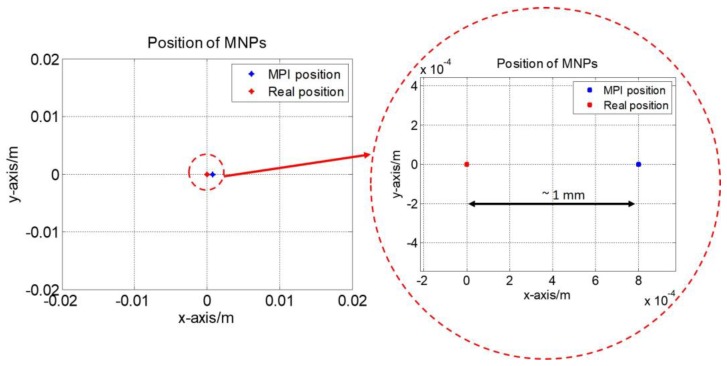
The difference between the MPI position and the real sample position when MNPs are at the center.

**Figure 7:**
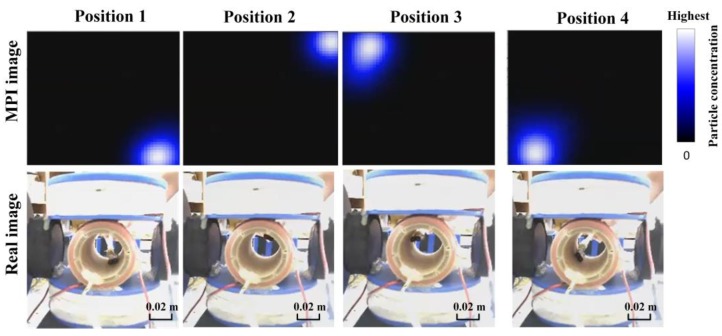
The Real-time 2D MPI image generation for tracking MNPs in the targeted drug delivery platform. The white color represents the maximum particle concentration and the black color shows the absence of particles.

**Figure 8:**
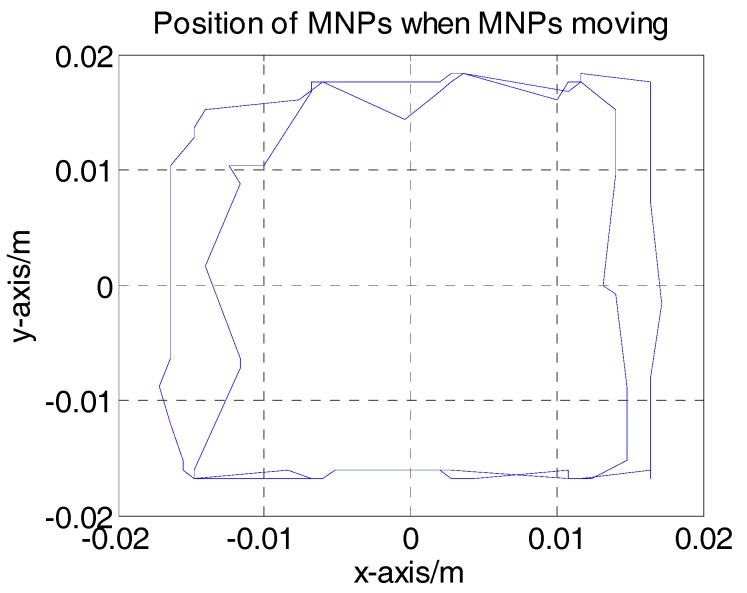
The trajectories of MNPs obtained from data of Figure 7.

Figure 6 shows the position of the MNPs at the center of workspace and the position of the center of the MPI image for the sample of MNP, respectively. In this experiment, the difference is less than 1 mm, which indicates an image resolution of 1 mm. To demonstrate the functionality of the real-time 2D monitoring system, the MNP position in the *x*-*y* plane is changed sequentially to right-side top, left-side top and left-side bottom. The update rate (2 Hz) enables the MPI image to track these changes and follow the particles in the 2D plane in real-time. The Hz rate (0.5 s) provides higher update rate compared to the MRI (1 s–1 h), [26]. An optical camera is used to capture the real position of the particles; a screenshot of the 2D AM MPI at the same time is provided in Figure 7. Figure 7 shows the sequential changes in particle location; the MPI responds to these changes in real-time. The MNP positions obtained by real-time MPI monitoring of the data in Figure 7 are shown in Figure 8.

The results in Figure 7 and Figure 8 show that the monitoring system can track MNPs in a 2D plane, which facilitates steering of MNPs. The update rate of 0.5 s achieved by the AM MPI monitoring scheme is feasible for TDD systems, and the platform could be used in a control loop for MNPs steering. Moreover, the 2D monitoring system could easily be expanded to a 3D system by adding one set of drive coils. By choosing currents of appropriate frequency in one set of coils, a 3D Cartesian trajectory can be achieved to cover a 3D workspace, and the received signal can be reconstructed to a 3D MPI image. 

### 4.3. Discussions for the Developed 2D MPI System

In this paper, the magnetic gradient *G_x_* in the *x*-axis was ~2.8 T/m/μ_0_ and the magnetic gradient *G_y_* in the *y*-axis was ~1.4 T/m/μ_0_. The resolution of MPI is proportional to the magnetic gradient strength and proportional to the cube of the particle’s diameter [34]. So, based on [34] with Resovist particles (5 nm core size), the theoretical intrinsic resolution in the *x*-direction should be larger than 7.9 mm, and the intrinsic resolution in the *y*-direction should be larger than 16 mm [34]. However, subsequent studies showed that the Resovist particles aggregate with an effective diameter of 24 nm for the iron core during MPI process [35]. Based on this diameter considering the aggregation, theoretic resolution value can be reaching to 1mm, whose value is matching to the value from the experiment result in Figure 6. 

The current system presented in this paper is suitable for mouse experiments (region of interest ROI of 60 mm × 100 mm) and it cannot be directly scaled up for human. For a scale up to accommodate a human scanner, as presented in [20], the highest feasible gradient strength which can be generated by a coil system is about 3 T/m/μ_0_. A recent study of MPI for human brain shows that using even magnetic field gradient of 1.5 T/m/μ_0_ for the human brain can provide a spatial resolution comparable to a FMRI image [36]. With the magnetic gradient of 3 T/m/μ_0_ and Resovist particles, the MPI can achieve a enough resolution, which can be applied to many applications [21]. 

To extend the proposed system to a human brain scale with the workspace of 250 mm × 250 mm, the diameter of coils should be increased at least twice times to reach a magnetic field gradient of about 3 T/m/μ_0_ for the MPI mode. The power and voltage of supplies also should be increased about four times. Although the condition of a static magnetic field is still safe for human [37], the magnetic gradient can represent a potential health risk beside peripheral nerve and cardiac stimulation to the patient [38]. Even though the required high power supply is available with today’s hardware technology, cooling issue, packaging, and safety issues should be considered for scalability of a human brain. In results, the MPI system developed for use on humans is still a big challenge today.

## 5. Conclusions

Over the past decade, MNPs have emerged as pioneering drug carriers for TDD. One of the main challenges in TDD with MNPs is real-time monitoring of the MNPs for precise drug delivery and analysis. Therefore, the feedforward approach is currently being utilized in most micro/nano robotic platforms. MPI-based platforms can provide a cost-efficient, compact, and high targeting rate for TDD. 

This paper provides valuable data that will facilitate the enhancement of drug delivery systems using real-time monitoring. Our new techniques can be adapted to drug delivery platforms for MNP steering. Future work should address the design and implementation of 2D real-time navigation systems. The systems should be scaled-up for the workspace and real-time in vivo imaging of MNPs should be performed to verify the utility of the proposed approach. Finally, the future works will implement the system feasible for TDD to the brain. 

## Figures and Tables

**Table 1:** Geometrical specifications of the coils in the 2D MPI system.

	Turns	Inner Diameter	Outer Diameter	Coil Length	Wire
Actuation/selection-drive coil (*x*-axis)	5000	0.070 m	0.190 m	0.070 m	1 mm copper wire
Actuation/drive coil (*y*-axis)	260	0.040 m	0.126 m	0.030 m	Litz wire
Receive coil	400	0.042 m	Single layer	0.060 m	Litz wire
Cancellation coil	200	0.042 m	Single layer	0.015 m	Litz wire
Excitation coil	44	0.050 m	Single layer	0.110 m	Litz wire

## Supplementary Materials

Supplementary File 1
